# Supplementary material for: Optimization of Vero Cells Grown on a Polymer Fiber Carrier in a Disposable Bioreactor for Inactivated Coxsackievirus A16 Vaccine Development
Source: Vaccines (Basel). 2021 Jun 7;9(6):613. doi: 10.3390/vaccines9060613 (PMC8229131; doi:10.3390/vaccines9060613)
Supplement: Supplementary file 1 [file vaccines-09-00613-s001.zip › vaccines-1197140-supplementary.pdf]

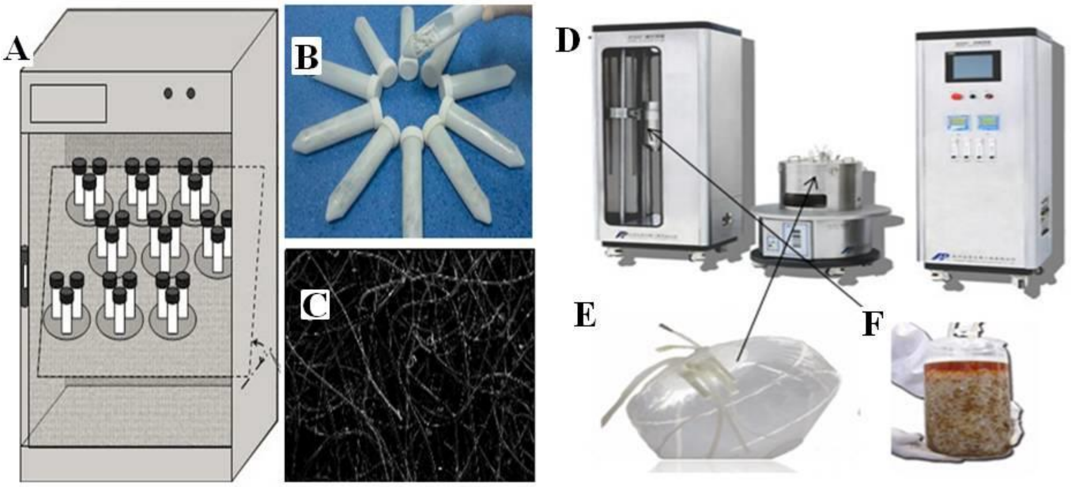

Figure S1. Illustration of the mini-bioreactor and ACPB system. **(A)** Self-rotating incubator with vessels. (**B)** Vessels packed with a polymer fiber carrier. **(C)** Photomircograph (200×) of polymer fiber carrier. (**D)** AmProtein Current Perfusion Bioreactor. (**E)** 5 L disposable plastic perfusion column. (**F)** 10 L disposable plastic bioreactor bag with DO, pH, and temperature probes.
